# Respiration and circulation affected by gas leakage into the abdominal cavity during endoscopic esophageal submucosal dissection after gastrostomy: a case report

**DOI:** 10.1186/s40981-021-00492-2

**Published:** 2022-01-08

**Authors:** Jun Honda, Keisuke Kuwana, Saori Kase, Shinju Obara, Satoki Inoue

**Affiliations:** 1 Department of Anesthesiology, Fukushima Medical University Hospital, 1 Hikarigaoka, Fukushima, 960-1295 Japan; 2 Surgical Operation Department, Fukushima Medical University Hospital, 1 Hikarigaoka, Fukushima, 960-1295 Japan

**Keywords:** Percutaneous endoscopic gastrostomy, Endoscopic submucosal dissection, Pneumoperitoneum, Insufflation gas leakage

## Abstract

**Background:**

Pneumoperitoneum is a common complication of percutaneous endoscopic gastrostomy (PEG). We report a case of circulatory and respiratory depression due to pneumoperitoneum caused by PEG dislodgement during endoscopic submucosal dissection (ESD) surgery.

**Case presentation:**

A 46-year-old man with PEG for dysphagia underwent ESD for esophageal cancer under general anesthesia. The patient developed a gradual increase in peak inspiratory pressure, followed by a decrease in peripheral oxygen saturation (SpO_2_) and blood pressure, as well as an increase in heart rate (HR) during endoscopic submucosal ESD for esophageal cancer. We suspected mediastinal emphysema due to esophageal perforation, but the surgery was successfully completed. Postoperative computed tomography (CT) revealed that the abdominal and gastric walls, which had been fixed by PEG, were detached, resulting in a large amount of intra-abdominal gas and mediastinal emphysema.

**Conclusions:**

ESD in patients with PEG should be performed carefully because of the possibility of intraoperative PEG dislodgement and pneumoperitoneum caused by insufflation gas leakage.

## Background

Percutaneous endoscopic gastrostomy (PEG) has been a common method of mid- to long-term enteral nutrition since its introduction in 1980.

Pneumoperitoneum is a common complication of the PEG procedure and does not require intervention in the absence of clinical symptoms [1], but the incidence of pneumoperitoneum is as high as 50% after prolonged PEG [2]. The cause of pneumoperitoneum in PEG patients is thought to be air leakage, probably due to inadequate fixation of the gastric wall [3]. In addition, when PEGs are dislodged, the anterior gastric wall and abdominal wall may separate, allowing gastric contents to leak into the abdominal cavity, resulting in peritonitis, which may require laparotomy [1].

In this report, we present a case of a PEG patient who underwent endoscopic submucosal dissection (ESD) under general anesthesia and experienced circulatory and respiratory depression due to accidental PEG bumper dislodgement and pneumoperitoneum, probably caused by insufflation gas leakage from the surgical field.

We obtained written informed consent from the patient to present this case.

## Case presentation

**Fig. 1:**
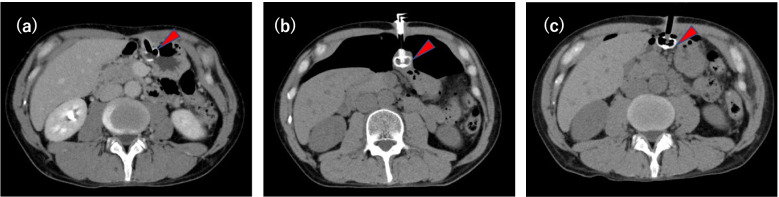
Computed tomographic images. Preoperative computed tomographic scans (**a**) showed percutaneous endoscopic gastrostomy bumper (red arrow) fixing stomach properly. Postoperative computed tomographic scans (**b**) showed percutaneous endoscopic gastrostomy bumper (red arrow) dislodged from abdominal wall. Postoperative day 4 computed tomographic scans (**c**) showed percutaneous endoscopic gastrostomy bumper (red arrow) protrude from stomach

**Fig. 2:**
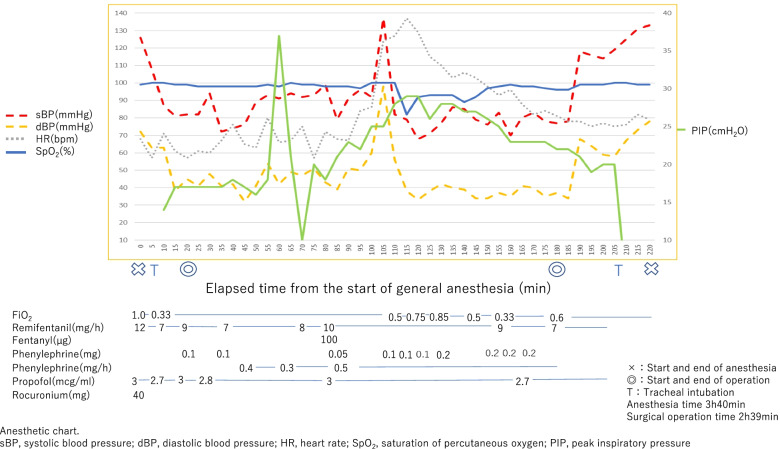
Anesthesia chart. sBP, systolic blood pressure; dBP, diastolic blood pressure; HR, heart rate; SpO2, saturation of percutaneous oxygen; PIP, peak inspiratory pressure

**Fig. 3:**
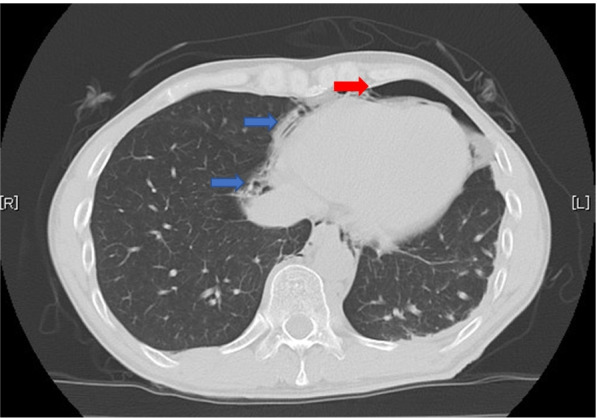
Computed tomographic images. Thoracic computed tomography showing a left-sided pneumothorax (red arrow) and mediastinal emphysema (blue arrow)

A 46-year-old man (185 cm in height, and 62 kg in weight) was scheduled for esophageal ESD under general anesthesia with tracheal intubation in the left lateral position. He had a history of radiochemotherapy for oropharyngeal cancer, and of PEG tube placement for dysphagia 6 month before (Fig. 1a). Anesthetic chart is shown in Fig. 2. General anesthesia was induced with propofol, remifentanil, and rocuronium followed by orotracheal intubation uneventfully. The ventilator was set in volume-controlled ventilation mode (FiO_2_ 0.35, TV 500 ml, RR 11/min, and PEEP 5 cm H_2_O). Gradual increase in peak inspiratory pressure occurred 60 min after starting surgery, which was increased from 18 cm H_2_O to 28 cm H_2_O in 30 min. An abrupt increase of systolic blood pressure from 90 to 134 mmHg, followed by a decrease in peripheral oxygen saturation (SpO_2_) from 100 to 82% occurred approximately 90 min after starting surgery. At this point, the surgeon told us that there was no possibility of esophageal perforation, but we suspected the possibility of mediastinal emphysema because of the subcutaneous emphysema in the anterior chest. SpO_2_ was increased to 92% and hemodynamic condition was subsided by increasing the inhalational oxygen concentration and the repeated dose of phenylephrine. Surgery completed successfully and the intubation tube was removed in the operating room. No intraoperative patient movement was observed. Postoperative computed tomography (CT) revealed pneumoperitoneum with dislodgement of the PEG tube (Figs. 1b, 3) After the PEG button was opened, the intra-abdominal gas decreased, but on the fourth postoperative day, the PEG bumper was dislodged from the stomach into the abdominal cavity and was removed (Fig. 1c). The fistula in the gastric wall closed spontaneously. Antibiotics were used to prevent intra-abdominal infection, and the patient was discharged 16 days after surgery as there were no signs of infection.

## Discussion

We reported pneumoperitoneum due to PEG bumper dislodgement during ESD in a PEG patient. We initially suspected mediastinal emphysema caused by esophageal perforation because of the sudden intraoperative changes in circulatory and respiratory status. However, postoperative CT showed PEG bumper dislodgement and a large amount of intra-abdominal gas accumulation. We concluded that it was highly likely that the insufflation gas in the surgical field had accumulated in the stomach, leaked into the abdominal cavity through the gastrostomy hole, and caused mediastinal emphysema via the esophageal hiatus. We believe that this increase in insufflation pressure may have exacerbated the patient’s respiratory depression. Pneumoperitoneum is a complication that occurs in as many as 50% of patients after PEG placement [2], but to our knowledge, there are no reports of PEG bumper dislodgement and pneumoperitoneum during esophageal ESD in PEG patients. Complications arising from CO2 insufflation include pneumothorax, mediastinal emphysema, and subcutaneous emphysema [4]. Pathways of CO2 gas leakage from the abdominal cavity to the thoracic cavity include surgically induced medically induced fistulae and diaphragmatic anatomy (e.g., esophageal hiatus and vena caval foramen) [5].

In the case of laparoscopic surgery, factors that increase the likelihood of subcutaneous emphysema, pneumothorax, and mediastinal emphysema include the flow rate and pressure of the delivered gas, and the gas usage should be recorded [6].

Several improvements can be made: First, in endoscopic esophageal surgery, we believe that the flow rate and pressure of the insufflation gas as well as the amount of gas used should be recorded as in laparoscopic surgery, because in PEG patients, air accumulation in the stomach may cause the PEG bumper dislodgement, resulting in pneumoperitoneum.

Second, intraoperative measures such as keeping the PEG button open or inserting a tube for degassing from the PEG may be useful to prevent PEG bumper dislodgement by reducing the increase in intragastric pressure.

Third, it is important to remember that there is a possibility that the insufflation gas leaked into the abdominal cavity when the PIP, HR, and end-tidal CO2 (ETCO2) began to rise. In esophageal endoscopy, it is advisable to consider the possibility of subcutaneous emphysema, pneumothorax, or mediastinal emphysema when PIP, HR, and ETCO2 are elevated.

## Conclusion

We experienced a case of circulatory and respiratory depression during ESD under general anesthesia due to pneumoperitoneum possibly caused by insufflation gas leakage in a PEG patient. Clinicians have to keep in mind the possibility of insufflation gas leakage when performing surgical procedures in PEG patients.

## Data Availability

Not applicable.

## Abbreviations

CO2: Carbon dioxide
CT: Computed tomography
ESD: Endoscopic submucosal dissection
ETCO2: End tidal carbon dioxide
HR: Heart rate
SpO2: Peripheral oxygen saturation
PEG: Percutaneous endoscopic gastrostomy
PIP: Peak inspiratory pressure
VCV: Volume-controlled ventilation
